# Machine Learning for Prediction of Tuberculosis Detection: Case Study of Trained African Giant Pouched Rats

**DOI:** 10.2196/50771

**Published:** 2024-04-16

**Authors:** Joan Jonathan, Alcardo Alex Barakabitze, Cynthia D Fast, Christophe Cox

**Affiliations:** 1 Department of Informatics and Information Technology Sokoine University of Agriculture Morogoro United Republic of Tanzania; 2 APOPO Rodent Project Sokoine University of Agriculture Morogoro United Republic of Tanzania; 3 Evolutionary Ecology Group, Department of Biology University of Antwerp Antwerp Belgium; 4 Rutgers Center for Cognitive Science Piscataway, NJ United States

**Keywords:** machine learning, African giant pouched rat, diagnosis, tuberculosis, health care

## Abstract

**Background:**

Technological advancement has led to the growth and rapid increase of tuberculosis (TB) medical data generated from different health care areas, including diagnosis. Prioritizing better adoption and acceptance of innovative diagnostic technology to reduce the spread of TB significantly benefits developing countries. Trained TB-detection rats are used in Tanzania and Ethiopia for operational research to complement other TB diagnostic tools. This technology has increased new TB case detection owing to its speed, cost-effectiveness, and sensitivity.

**Objective:**

During the TB detection process, rats produce vast amounts of data, providing an opportunity to identify interesting patterns that influence TB detection performance. This study aimed to develop models that predict if the rat will hit (indicate the presence of TB within) the sample or not using machine learning (ML) techniques. The goal was to improve the diagnostic accuracy and performance of TB detection involving rats.

**Methods:**

APOPO (Anti-Persoonsmijnen Ontmijnende Product Ontwikkeling) Center in Morogoro provided data for this study from 2012 to 2019, and 366,441 observations were used to build predictive models using ML techniques, including decision tree, random forest, naïve Bayes, support vector machine, and k-nearest neighbor, by incorporating a variety of variables, such as the diagnostic results from partner health clinics using methods endorsed by the World Health Organization (WHO).

**Results:**

The support vector machine technique yielded the highest accuracy of 83.39% for prediction compared to other ML techniques used. Furthermore, this study found that the inclusion of variables related to whether the sample contained TB or not increased the performance accuracy of the predictive model.

**Conclusions:**

The inclusion of variables related to the diagnostic results of TB samples may improve the detection performance of the trained rats. The study results may be of importance to TB-detection rat trainers and TB decision-makers as the results may prompt them to take action to maintain the usefulness of the technology and increase the TB detection performance of trained rats.

## Introduction

### Background

African giant pouched rats (*Cricetomys ansorgei*) are native to sub-Saharan Africa, making them resistant to local parasites and diseases [1]. The term “pouched rat” refers to their large cheek pouches that are used for carrying food back to their burrows, where the food is either eaten or stored. These rats are nocturnal and omnivorous, eating various insects, fruits, and vegetables. They are large (adult males and females weigh about 1.3 kg and 1.2 kg, respectively) and are long-lived, averaging 8 years in captivity. Moreover, they have a highly developed olfactory capacity, enabling them to do specific detection tasks with training [2]. As such, in 1997, APOPO (Anti-Persoonsmijnen Ontmijnende Product Ontwikkeling or “Anti-Personnel Landmines Detection Product Development” in English) started researching how to train these rats for scent detection. APOPO is a Belgian nongovernmental organization whose mission is to protect people and the planet using scent detection animals [3]. Rat pups born at APOPO’s breeding facility are weaned from their mother at 10 weeks old. Rats begin training in a custom-engineered line cage immediately after they are weaned. Training for tuberculosis (TB) detection takes place in this apparatus, which requires upwards of 9 months to master. Each rat’s home cage is outfitted with a clay nest pot to simulate the rat’s natural underground burrow, a wood shaving substrate, and unlimited access to water that is routinely infused with a multivitamin and electrolyte supplement. The majority of the diet of the rats is provided during training sessions in the form of crushed commercial rodent chow pellets mixed with mashed bananas and avocados, which serves as appetitive reinforcement for the operant conditioning procedures. This diet is supplemented with a variety of fresh fruits, vegetables, and grains [3].

While APOPO began with training rats to detect landmines in former conflict zones, the demonstrated success influenced the 2001 idea to also train the rats to detect the presence of Mycobacterium tuberculosis in human sputum samples [4]. Data reported annually to the World Health Organization (WHO) by countries show that TB is one of the major causes of ill health and death worldwide. TB is a life-threatening infectious disease that attacks the lungs and can also harm other parts of the body. The transmission occurs from one person to another when a person with TB talks, sneezes, or coughs. The development of novel, accurate, robust, and rapid diagnostic capabilities will result in improved case detection, disease surveillance, health care delivery, and quality of future research [5]. In 2004, APOPO and Sokoine University of Agriculture (SUA) partnered with the Tanzanian National Institute of Medical Research (NIMR) and the Tanzanian National Tuberculosis and Leprosy Program (NTLP) to develop a scent-detection technology for diagnosing human TB in resource-poor areas [6]. While microscopy is the most commonly used method to detect TB in developing countries, its effectiveness remains a problem [3]. In Tanzania, the Ministry of Health, Community Development, Gender, Elders, and Children (MOHCDGEC) permitted APOPO to conduct research using rats to detect TB bacteria in sputum samples [7].

**Figure 1:**
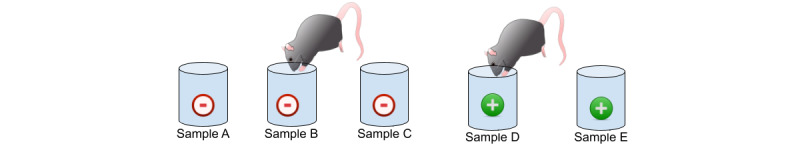
Tuberculosis (TB) testing and detection using trained rats. The rats test and detect TB-negative and TB-positive samples.

Figure 1 illustrates the concept of rat scent detection of TB. Sputum samples collected from partner DOTS (directly-observed treatment, short-course) clinics are heat inactivated (autoclaved) and then loaded into aluminum bars, which are positioned beneath holes in the floor of the line cage apparatus [4]. The rat sniffs each sample in succession as it walks from one side of the apparatus to the other. The rats are trained to pause over TB-positive samples for about 3 seconds but to quickly move past TB-negative samples [1]. During operational research, rats are rewarded with food for correctly pausing over (or “indicating”) samples that the DOTS clinic has determined to be TB positive. Samples which the DOTS clinic determines to be TB negative but which the rat indicates as TB positive (by pausing for 3 seconds) are flagged as suspect and subjected to additional confirmatory diagnostics in APOPO’s laboratory, using WHO-endorsed methods (typically, concentrated smear microscopy). During routine operational research, APOPO’s scent detection rats evaluate upwards of 100 samples (averaging 10% TB positive) from DOTS clinics within each 20-minute session. Referencing sample and patient information within a secure database allows APOPO to immediately notify the DOTS clinic of new cases so the patient can be contacted and can begin treatment. This procedure has effectively identified more than 29,000 TB patients who had a missed diagnosis prior to evaluation by TB-detection rats [4].

### Conceptual Framework

The theoretical concepts and empirical framework of this study are based on Signal Detection Theory (SDT). SDT describes how features of the stimulus and detector factors affect performance on stimulus detection tasks [8]. SDT helps to distinguish between the sensitivity of a detector and the underlying signal. In medical diagnosis, this translates to the efficacy of a diagnostic tool to accurately detect the presence of a pathogen or other signal with medical significance [9], that is, the diagnostic “sensitivity.” However, in rats, determining diagnostic accuracy depends on the rat’s training and the diagnostic results from partner health clinics using WHO-endorsed methods. During training, the behavior of each rat is recorded, including indication responses committed in response to samples known to either contain or not contain TB (TB positive or TB negative). These data allow trainers to accurately track each rat’s discrimination learning [4]. There are numerous independent variables related to each rat evaluation session, including the rat’s identity (name), age, sex, and bodyweight, as well as the characteristics of the sample itself, including DOTS clinic diagnostic results (ID_BL_DOTS) and results of any applicable confirmatory diagnosis within APOPO’s laboratory (ID_BL_APOPO), which are combined to form another independent variable called TB_Status.

**Figure 2:**
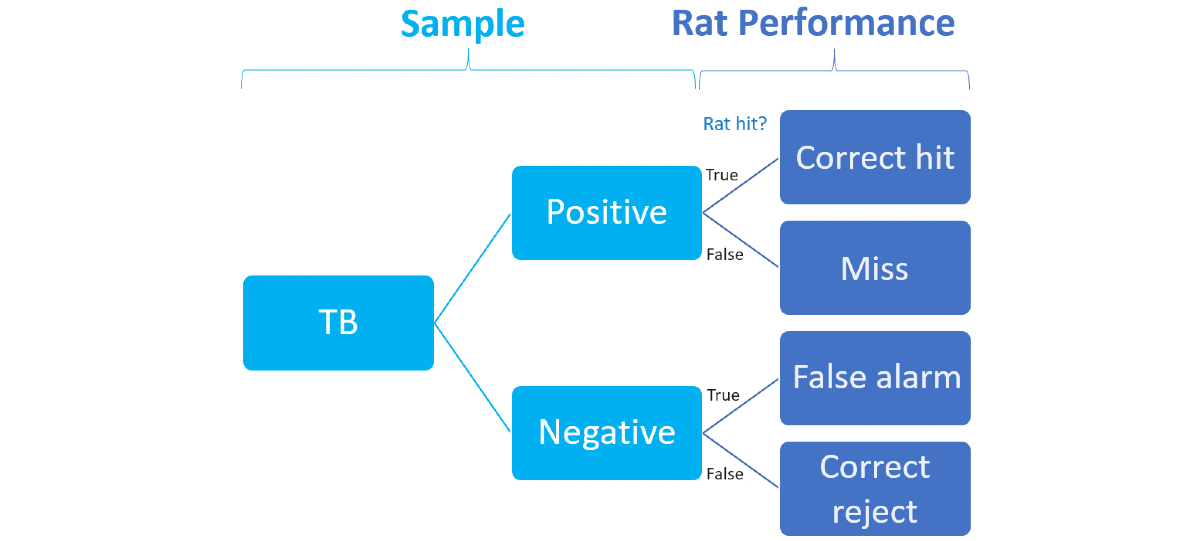
Relationship among the status of tuberculosis (TB), hit, and the performance of the rat. Hit refers to whether or not (true or false) the rat provided an indication.

In this study, one of the primary dependent variables was captured as hit, which refers to whether or not (true or false) the rat provided an indication (continuously sniffed the sample for at least 3 seconds, as estimated by the rat handler). Combining the hit variable with WHO-endorsed diagnostic results (ID_BL_DOTS and ID_BL_APOPO) provided 4 possible outcomes termed rat performance for each sample evaluated (Figure 2), including correct hit, miss, false alarm, and correct reject, which are used in determining the diagnostic accuracy of each rat. Correct hit refers to samples that the rat indicated and were confirmed to contain TB; false alarm (or suspect) refers to samples that the rat indicated but which could not be confirmed to contain TB. Additionally, miss (sample confirmed to be TB positive) and correct reject (no TB mycobacterium confirmed) refer to samples that the rat failed to indicate (sniff for 3 seconds) [3]. In other words, the rat’s sensitivity represents the percentage of correct hits out of the sum of total correct hits and total misses (all confirmed TB-positive samples evaluated by the rat). Similarly, the rat’s specificity represents the percentage of correct rejects out of the sum of correct rejects and false alarms (all samples found to be TB negative) [10]. By this logic, sensitivity refers to the rat’s ability to accurately find true positive (TP) cases, while specificity measures its ability to accurately reject negative cases. Hence, sensitivity (correct hit) and specificity (correct reject) together comprise overall diagnostic accuracy.

From Figure 2, if the TB status was already known to be positive at the time of the rat evaluation and hit was true, the rat’s behavior was categorized as “correct hit.” Conversely, if the TB status was positive and hit was false, the rat’s behavior was categorized as “miss.” On the other hand, if the TB status was determined to be negative at the time of the rat evaluation and hit was true, the rat’s behavior was categorized as “false alarm” or suspect. Finally, if the TB status was negative and hit was false, the rat’s behavior was categorized as “correct reject.”

Hence, contrary to the study by Jonathan et al [10], this study considered the status of TB in the sample the rat was evaluating. In that study, the modeling methods only used the dichotomous variable of hit as true or false (ie, did the rat sniff the sample for ≥3 seconds) without regard for what the rat was sniffing. Within the data set analyzed, about 78.8% of samples were not hit (hit=false), somewhat reflecting the estimated underlying prevalence of TB across the samples. However, assuming this distribution reflected that the most common outcome (hit=false) served as the desired or correct outcome in all instances when modeling rat performance, the models predicted when a trained rat would fail to detect TB (ie, miss a TB-positive sample or correctly reject a TB-negative sample) rather than detect it. Furthermore, the predictive power of the models did not take into account what the rats were smelling, since the rats were trained to perform differently (hit true or false) depending on the presence of TB within the sample. Therefore, the aim of this study was to replicate the procedures of the study by Jonathan et al [10] but with the inclusion of variables related to the detection of TB and with expansion of modeling to include 2 additional machine learning (ML) algorithms.

### Objectives of the Study

This study applied the same data set from APOPO’s TB-detection rat training and research center in Morogoro, Tanzania, as used by Jonathan et al [10] but with the inclusion of WHO-endorsed diagnostic results, including those provided by partner DOTS clinics (smear microscopy, ID_BL_DOTS) and, where applicable, those performed by APOPO (either concentrated smear microscopy, ID_BL_FM, or fluorescent microscopy, ID_BL_APOPO) to confirm samples flagged suspect by the rats. As with Jonathan et al [10], this study used the decision tree, random forest, and naïve Bayes algorithms and included support vector machine (SVM) and k-nearest neighbor (kNN) ML techniques to improve the accuracy of the predictive models. Furthermore, it provides extensive simulations using real data to determine if ML techniques can accurately predict the performance of rat TB detection. Additionally, this paper compares the classification accuracy performance of the 5 ML predictive models. The rest of this paper is organized as follows: the Related Work subsection provides details of related literature focusing on African giant pouched rat TB detection, including the current status and its implications, along with the application of ML in diagnosing and detecting TB; the Methods section presents the methodology of this study; the Results section provides a description of the performance results and performance measurements of the predictive models; and the Discussion section discusses the findings, provides conclusions, and mentions the scope for future work.

### Related Work

#### Diagnosis of TB by African Giant Pouched Rats: Current Status and its Implications

African giant pouched rats cost-efficiently complement other TB diagnostic tools through second-line screening via scent detection to increase TB case detection. Patient samples are provided by partner DOTS clinics that perform initial screening. The rats can test up to 100 samples in 20 minutes or less, while a laboratory technician requires about 4 days to accomplish the same task using microscopy [11]. Samples that the clinic deems TB negative but which the rats indicate are TB positive are then retested using WHO-endorsed methods, such as concentrated smear microscopy or GeneXpert. Samples that are confirmed positive are communicated to the respective DOTS clinic, effectively providing 24-hour result turnaround and improved linkage to care [6]. Applying this method since 2007 has enabled TB-detection rats to identify more than 29,000 patients who had a missed diagnosis during initial screening [4]. Thus, rat scent detection technology is of great importance to the community and public health hospitals because it increases case detection, enables treatment, and curbs the spread of the disease [3].

#### Application of ML and Big Data Analytics in Diagnosing and Detecting TB

Technology advancement has allowed access to data from multidimensional sources with high throughput velocity. The term used to describe this kind of data is “big data,” which is difficult to analyze for interesting patterns or inefficiencies without ML technologies [12]. The application of ML in health care is important to improve human health, and ML and big data analytic technologies have brought advancements in TB health care services owing to the increase of health care data and the availability of analytics to solve health problems [13]. ML is a technology that enables a machine to learn from past data and predict the outcome. Thus, in health care, ML contains sophisticated algorithms that help to learn features from a large volume of health care data and then use the obtained insights to assist clinical practices [14]. Big data analytics is the use of advanced analytic techniques on vast amounts of data in different formats, such as structured, semistructured, and unstructured data, from different sources. Big data analytics can help to discover useful information that facilitates decision-making and health care outcome prediction. Therefore, ML and big data analytics can assist physicians by providing up-to-date medical information from clinical practices for proper patient care. As such, the application of ML and big data analytics can help to reduce diagnostic and human errors in the outcomes of clinical practices [15].

ML in health care depends on different techniques, which include classification, clustering, and association, for its operation. These techniques help to learn past data and detect knowledge patterns [16]. Classification techniques are used to develop models that predict future events from the manipulated data and offer solutions to real-world health problems such as diagnosis and treatment of diseases [16]. Classification is the ML technique that operates by building predictive models that categorize and assign labels to manipulated and newly encountered instances [16]. These predictive models help solve multiclassification problems through prediction and analysis. Moreover, the models are used as decision-support tools that help medical professionals interpret diagnosis results [17]. For example, Abdar et al [18] used the boosted C5.0 and CHAID classification algorithms to build a decision tree model for the early diagnosis and prediction of liver disease. In addition, ML technologies were used in the diagnosis of TB to categorize and find relationships among the manipulated variables [19]. This study developed an efficient and reliable framework for automatic TB bacilli detection based on deep learning and ML algorithms. The study also suggested that a classification model can be used to discriminate between positive and negative samples [19].

The classification algorithms recently used in the diagnosis of TB include decision tree, random forest, naïve Bayes, SVM, and kNN [20]. These algorithms are suggested as an alternative for health care professionals to improve the diagnosis of TB. The decision tree algorithm C4.5 was used to build a model to predict the presence of TB bacteria. The results showed that the decision tree had a prediction accuracy of 99% [21]. The decision tree generates rules that are simple and easy to understand and interpret for a decision maker [16].

Moreover, a random forest classification algorithm was used to discriminate the TB bacilli with a sensitivity and specificity of above 89.34% and 62.89%, respectively. Furthermore, it is proposed that the naïve Bayes algorithm can be used for the diagnosis of TB [22]. Additionally, SVM is known as a useful model to identify abnormalities in the lungs for the diagnosis of TB [23]. Following this, algorithm comparison is of great importance to find a reliable algorithm in the given data [24].

## Methods

### ML Algorithms

In this study, the ML algorithms used are decision tree, random forest, naïve Bayes, SVM, and kNN to build predictive models that categorize data and assign a label to manipulated and newly encountered data. The purpose of involving different algorithms is to compare and improve the prediction accuracy of rats for TB detection.

### Real Data Sets

This paper used 2 data sets provided by APOPO: detection rats data set and RAT_WEIGHT data set, which were combined to form the final data set, as shown in Table 1. The detection rats data set contained 471,133 observations from 2011 to 2019 and involved 18 variables (17 independent and 1 dependent). The RAT_WEIGHT data set contained 1438 records collected from 2012 to 2019 and involved 4 independent variables. Moreover, these data contained 5 female rats with IDs 56, 72, 80, 85, and 96. However, the fifth rat with ID 96 from the RAT_WEIGHT data set was eliminated in the analysis because it lacked the necessary detection performance variables in the detection rats data set. Therefore, 4 female rats were used in this study. The 2 data sets and corresponding variables are displayed in Table 1.

**Table 1:** Rats data set description.

Data set and number		Variable name	Data type	Description	Variable type
**Detection rats data set**					
	1	DOTS_NAME	String	Name of the DOTS^a^ center	Independent
	2	DOTS_PATIENTS_NUMBER	Integer	Number of patients from the DOTS center	Independent
	3	ENTRY_YEAR	Integer	Year when the patient attended the DOTS center	Independent
	4	ID_SAMPLE	Integer	Identification of the sample	Independent
	5	ID_BL_DOTS	Integer	Identification of the bacteria level from the DOTS center	Independent
	6	HIT	Boolean	TB^b^ detection rat performance (categorical variable)	Dependent
	7	ID_BL_APOPO	Integer	Identification of the bacteria level from the APOPO^c^ center	Independent
	8	ID_CONFIGURATION	Integer	Identification of the cage during training	Independent
	9	ID_BL_FM	Integer	Identification of the bacteria level by fluorescence microscopy	Independent
	10	ID_EVALUATION_SESSION	Integer	Identification of the evaluation session	Independent
	11	SESSION_DATE	Date	Date when a session was performed	Independent
	12	ID_RAT	Integer	Identification of the rat	Independent
	13	RAT_NAME	String	Name of the rat	Independent
	14	GENDER	String	Sex of the rat	Independent
	15	AGE	Integer	Age of the rat	Independent
	16	START_TIME	DateTime	Date and time when the detection task started	Independent
	17	END_TIME	DateTime	Date and time when the detection task ended	Independent
	18	DOB	Date	Date when the rat was born	Independent
**RAT_WEIGHT data set**					
	1	ID_RAT	Integer	Identification of the rat	Independent
	2	RAT_NAME	String	Name of the rat	Independent
	3	WEIGHT_DATE	Date	Date when the weight of the rat was measured	Independent
	4	WEIGHT	Integer	Weight of the rat	Independent

^a^DOTS: directly-observed treatment, short-course.

^b^TB: tuberculosis.

^c^APOPO: Anti-Persoonsmijnen Ontmijnende Product Ontwikkeling.

### Applied Variables

The data underwent initial preprocessing to obtain the required variables for developing the predictive models. All data preparation was implemented by Python owing to its large number of libraries for scientific computing and the development of ML predictive models [24]. The sample (either TB negative or the bacterial concentration of TB positivity provided by the partner DOTS clinic, ID_BL_DOTS) was compared to APOPO’s confirmatory diagnosis (where applicable) using concentrated smear microscopy (ID_BL_APOPO) to create a variable termed Definitive_Status. This variable reflected the APOPO result when one was provided; otherwise, it indicated the DOTS clinic result. The Definitive_Status was then transformed into the dichotomous variables of TB_Status to reflect the final status of the sample as either positive or negative for TB (collapsing across bacterial concentrations for positive samples). Then, TB_Status was compared to hit to compute the dependent variable of Rat_Performance, which consists of 4 categories: correct hit, miss, false alarm, and correct reject (Figure 2).

After the data preparation, 4 variables for the detection performance of the rats, including TB_Status, age, weight, and hit, as shown in Table 2, were used to build the predictive model. Moreover, this study used 366,441 observations for analysis after removing the null rows from the rats data set to prevent noises, outliers, and inconsistencies in the data. The sklearn model selection library through a train-test split class was used to partition the data (366,441 observations) into 256,508 observations (70%) in the training data and 109,933 observations (30%) in the test data. It is important to mention that, due to the binary nature of many variables and the underlying prevalence of TB infections, the data used in this study lack a normal distribution, as shown in Multimedia Appendix 1.

Categorical variables were used to build predictive models, and 256,508 observations (70%) were used for training the models. The TB_Status variable consisted of 10.90% (27,950/256,508) positive samples and 89.10% (228,558/256,508) negative samples. The hit variable consisted of 21.33% (54,719/256,508) true values and 78.67% (201,789/256,508) false values.

Table 3 shows a statistical summary of the distribution of continuous variable data before and after the random data split. Despite most of the distributions being the same, the mean of age and weight variables showed a difference of 0.01. Moreover, the SD of ID_RAT and weight differed by 0.01.

**Table 2:** Description of the dependent and independent variables used to build predictive models.

Variable	Description	Data type	Variable type	Value
TB_Status	Final diagnosis of the sample as either TB^a^ positive or TB negative. Combines the diagnostic results of both DOTS^b^ and APOPO^c^ (lab confirmation, when applicable) wherein APOPO status (results) overrides DOTS.	Object	Independent/categorical	True or false
Age	Age of the rat in years at the time when the rat evaluated the patient sample in question	Object	Independent	Age ranges from 0.79 to 7.95 years
Weight	Average rat body weight (in grams) per year because most of DetectionRatsData describes the daily detection tasks and misses their corresponding weights since the weight of the rats from the RAT_WEIGHT data set was measured every week.	Object	Independent	Average rat body weight ranges from 843.67 to 1054.83 grams
Hit^d^	Defined as a continuous sniff (nose insertion into the cage hole) for ≥3 seconds. True means the rat “indicated” that the sample contained TB (held its nose in the hole for at least 3 seconds). False means the rat rejected the sample (did not hold its nose for at least 3 seconds).	Object	Dependent/categorical	True or false

^a^TB: tuberculosis.

^b^DOTS: directly-observed treatment, short-course.

^c^APOPO: Anti-Persoonsmijnen Ontmijnende Product Ontwikkeling.

^d^Hit refers to whether or not (true or false) the rat provided an indication.

**Table 3:** Descriptive statistics of the continuous variables used to build predictive models before and after random data split.

Data split status and variable		Age (years)	Weight (g)
**Before random data split (n=366,441, 100%)**			
	Mean	3.83	899.40
	SD	1.72	84.37
	IQR	3.71	866.80
	Minimum	0.79	843.67
	Maximum	7.95	1054.83
**After random data split (n=256,508, 70%)**			
	Mean	3.84	899.41
	SD	1.72	84.36
	IQR	3.71	866.80
	Minimum	0.79	843.67
	Maximum	7.95	1054.83

### Model Building

The predictive model in this study was developed using 5 different ML techniques: decision tree, random forest, naïve Bayes, SVM, and kNN. This study used Python libraries for data preprocessing, matrix processing, mathematical functions, visualization, and classification. These are Pandas, Numpy, Matplotlib, and Scikit-learn [25]. The repetitive approach was used to generate a decision tree by dividing the training data. The data were divided recursively until the same class of variables, depending on conditions, using roughly 15,000 samples per leaf, were distributed among each division to create the decision tree. After that, each node in the decision tree used a split point to test the altered variables and choose how to divide the data. The split decision was concerned with the information gain and entropy of a computed variable. The variable that had the greatest information gain and the least entropy was therefore divided and put to the test. The choice regarding the data split and decision tree building was made based on information gain and entropy [16]. This study used pruning to maintain control over the parameters being used to remedy expansion.

During the training procedure, many decision trees were randomly constructed using the random forest technique. Based on the provided manipulated variables, the algorithm’s ultimate decision was based on the selection of the majority of the trees. There was a connection between the outcome and the number of trees in the forest. The outcome was therefore more accurate with an increase in the number of trees. As a result, the technique handled 500 trees in the ensemble, and it calculated the error rate using the training set of information. In the random forest approach, the training data were used to generate random splits for the root node and variable node. Since there was no parameter control during training, the connection between trees remained strong. Additionally, the frequency and values of the adjusted variables from the provided data were counted to generate the classification model using the naïve Bayes method. This method determined the dependent variable’s a priori probabilities as well as the conditional probabilities for each independent variable based on the altered data. The naïve Bayes technique has been specifically utilized to contrast its prediction performance with the outcomes produced by other ML techniques. It does not display the weights of each variable included in the classification.

SVM is one of the most common supervised ML algorithms owing to its greater predictive power. SVM analyzes data, recognizes patterns, and produces input-output functions from a set of labeled training data. It works by classifying a response variable by drawing a decision boundary line or hyperplane to separate 2 classes. Then, the maximum margin hyperplanes are constructed to optimally separate the output classes from each other in the training data. The goal is to find the optimal separating hyperplane where the separating margin is maximized. The linear kernel was used to allow flexibility and loss functions. The kNN algorithm is a supervised ML algorithm that works by identifying a set of k-nearest observations to the test point and calculating mainly the Euclidean distance between an observation and its kNN in training data. The k in kNN refers to the number of nearest neighbors the classifier will retrieve and use to make its prediction. The chosen k in kNN was 1, as it is suggested to provide the best test prediction.

### Performance Measurements

This study used accuracy, specificity, sensitivity, and F1 score as metrics to evaluate the performance of the generated predictive models and compare classification performances. These measurements were supported in the *scikit-learn* library through the classification report class.

#### Accuracy

The classification accuracy was calculated based on the confusion matrix, which accurately categorized the actual class labels of the test data and the class labels of the predicted models. It was also obtained by dividing the number of truly classified instances by the number of instances in the test phase. Accuracy considers TP, true negative (TN), false positive (FP), and false negative (FN). The classification accuracy for the data set was measured according to the following formula:

Accuracy = (TP + TN) / (TP + FP + TN + FN) **(1)**

#### Sensitivity

Sensitivity is defined as the number of TP cases over the number of TP cases plus the number of FN cases. Sensitivity identifies the correct positive predictions relative to the total actual positive cases. It is sometimes called a recall metric. The formula of sensitivity is as follows:

Sensitivity = TP / (TP + FN) **(2)**

#### Specificity

Specificity is the ratio between TN cases and all negative cases. In this study, the precision measure identified the correct positive predictions relative to total positive predictions. For diagnostic tools, this could be termed positive predictive value (PPV) or precision. It essentially provides confidence that any given positive response reflects a truly positive condition [25]. The formula of specificity is as follows:

Specificity = TN / (TN + FP) **(3)**

#### F1 Score

The F1 score is the harmonic mean of specificity and sensitivity. Basically, it is the weighted average of specificity and sensitivity. The F1 score was calculated from the specificity and sensitivity of the test data set [25]. The formula of the F1 score is as follows:

F1 score = 2 ([Precision × Sensitivity] / [Precision + Sensitivity]) **(4)**

It is important to mention that specificity and sensitivity are similar to precision and recall, respectively.

### Restrictions of the Study

This study ran the predictive models on a computer with a Core i5-5300U CPU at 2.30 GHz (2301 MHz, 2 cores, 4 logical processors) and 8 GB of RAM. The sample size, on the other hand, was small, with only 4 rats and a gender imbalance. Moreover, the hit variable consisted of fewer true values (21.26%) than false values (78.74%).

### Ethical Considerations

The study was approved by the SUA (DPRTC/R/142/vol.01/104) and Medical Research Coordinating Committee of Tanzania (NIMR/HQ/R.8a/Vol.1X/3905). The use of African giant pouched rats as a potential tool for TB diagnosis has received ethics clearance from the Tanzanian Medical Research Coordinating Committee [26]. The Office of Laboratory Animal Welfare has approved APOPO’s Animal Welfare Assurance (OLAW; Assurance Identification Number A5720-01).

## Results

### Comparing Classification Performance Measurements of the Predictive Models

**Figure 3:**
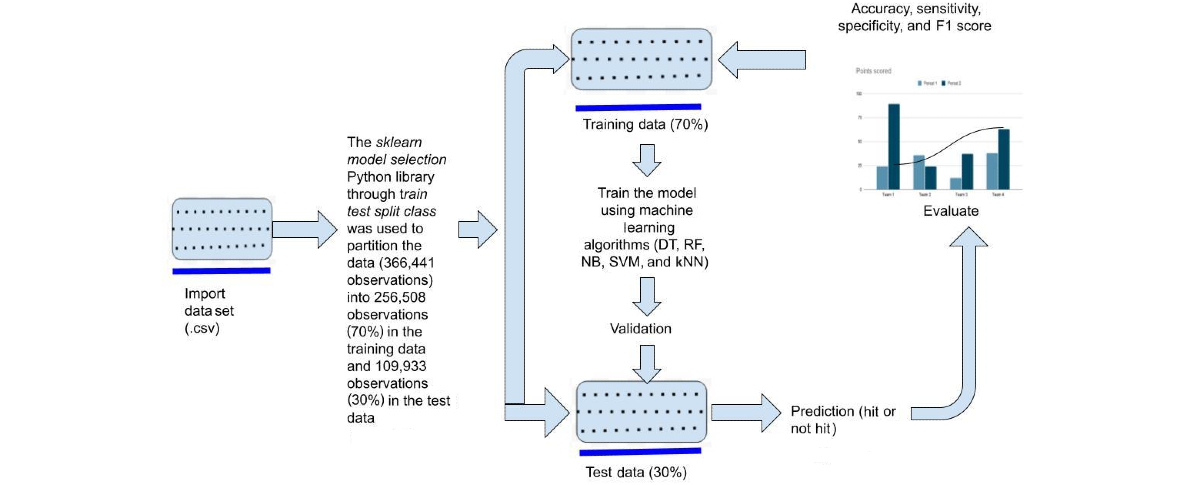
Process flow of machine learning–based prediction models of rat tuberculosis detection performance. The rectangle symbols represent data, while the histogram entails model evaluation metrics. DT: decision tree; kNN: k-nearest neighbor; NB: naïve Bayes; RF: random forest; SVM: support vector machine.

**Figure 4:**
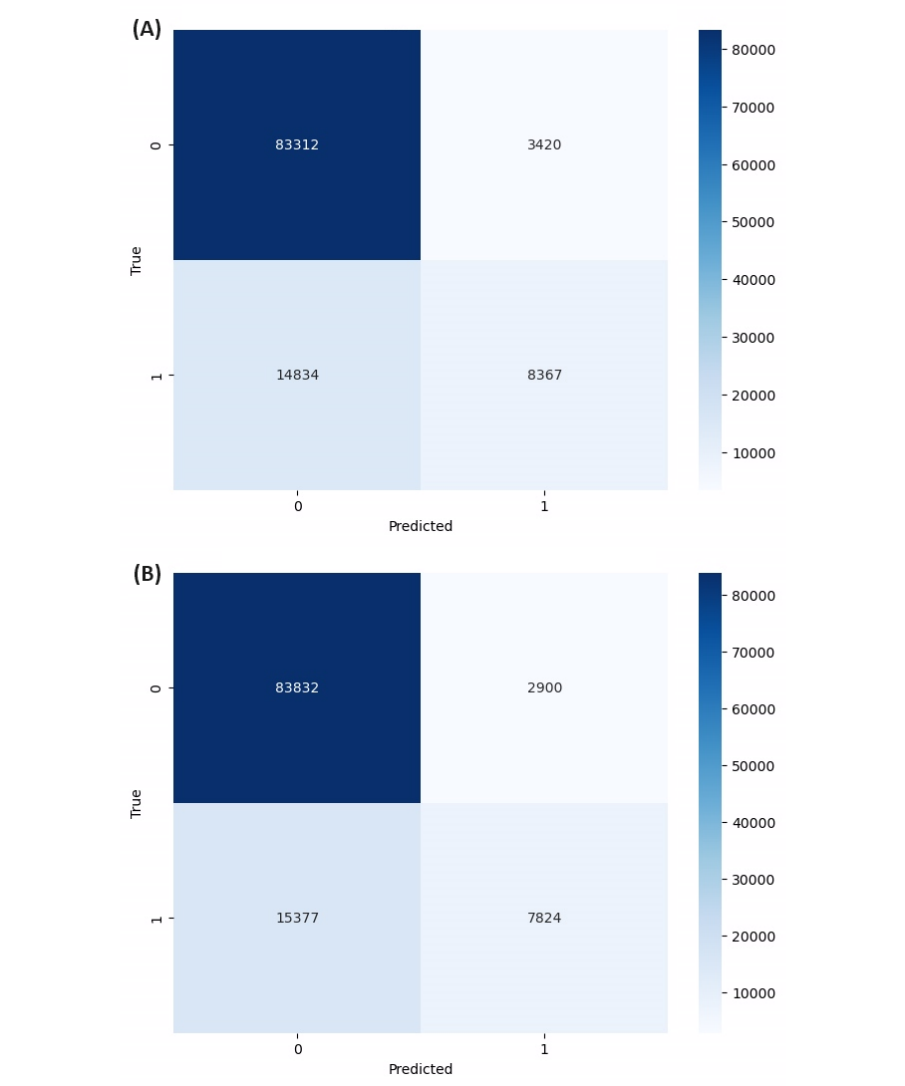
Confusion matrices of the predictive models. (A) Support vector machine classifier; (B) Random forest classifier.

This study used different ML techniques to build the predictive models following the methodology presented in Figure 3. Moreover, this study employed several metrics, including accuracy, sensitivity, specificity, and F1 score, to measure the classification performance of the predictive models based on test data. Figure 4 shows the confusion matrices of the SVM and random forest classifiers, while Table 4 summarizes the performance of all 5 ML techniques used to build the predictive models. The accuracy classification performance of the kNN technique was low at about 81.25%, while the best performing algorithm was SVM. As it can be seen from Table 4, validation showed that the SVM classifier based on the 4 variables shown in Table 2 achieved an accuracy of 83.39%, but it also reported that SVM had better ability to recognize the status of TB as either positive or negative in a given sample. 

**Table 4:** Comparing the classification performance of classifiers of rat tuberculosis detection.

Classification performance measurement	Predictive model				
	Decision tree	Random forest	Naïve Bayes	Support vector machine	K-nearest neighbor
Accuracy, %	83.32	83.38	82.56	83.39	81.25
Sensitivity, %	65.00	65.00	63.00	66.00	64.05
Specificity, %	79.00	79.00	77.00	78.00	72.05
F1 score, %	67.00	67.00	66.00	69.00	66.05
Correctly classified observations (true positive), n	91,602	91,602	90,370	91,602	89,326
Incorrectly classified observations (false negative), n	18,331	18,331	19,163	18,331	20,607

### Important Variables Influencing the TB Detection Performance of the Rats

This study used the random forest variable importance function to output the predictor variables based on the mean decrease in Gini (impurity). Random forest showed high performance in the feature ranking. The mean decrease in the Gini value is the average (mean) of a variable’s total decrease in the likelihood of incorrect classification of a new instance of a random variable from the data set. Multimedia Appendix 2 shows the predicted variable importance based on the mean decrease in the Gini value using the random forest algorithm.

From Table 5, higher (0.817152) and lower (0.026657) mean decreases in the Gini value result in greater and less variable importance, respectively. In other words, TB_Status and weight were the most and least significant variables, respectively, for predicting rat TB detection accuracy. However, for easy interpretation and visualization of these results, the variable importance function of the random forest algorithm sorted and displayed the variables as reported in Multimedia Appendix 2 based on the prediction importance. As such, the variable that contributed most to the prediction had the highest mean decrease in Gini values, followed by the variables with less importance.

**Table 5:** Random forest variable importance based on the mean decrease in the Gini value.

Variable	Variable name	Mean decrease in the Gini value
0	TB_Status	0.817152
1	Age	0.156190
2	Weight	0.026657

### Algorithm for the Prediction of Rat TB Detection Performance

The study also employed a prediction algorithm for TB detection as illustrated in Textbox 1.

Textbox 1 shows the algorithm that predicts if the rat will hit the sample or not. First, data were imported and normalized to acquire the required data format. Then, the statistical summary of the independent variables used to build predictive models was described. Considering Figure 3, the train_test_split library was used to divide the data set into training data (70%) for developing the models and test data (30%) for validating the models. The predictive models were trained based on the decision tree, random forest, naïve Bayes, SVM, and kNN classifiers, using the train data. Meanwhile, the validation of the models was performed using the test data. Then, accuracy, sensitivity, specificity, and F1 score were used to measure the classification performance of each classifier, as reported in Table 4. Furthermore, the input variables TB_Status, age, and weight were entered for prediction. Following the prediction, models were validated using the test data. Hence, data visualization was performed using the Matplotlib library for proper interpretation of the results. On the other hand, if the constraints were not met, the algorithm could be terminated.

In addition to the above algorithm for the prediction of rat TB detection performance, Figure 3 indicates the process flow of ML models and their predictions using *Python* libraries. The TB input data set was imported as a .csv file. After preprocessing the data, the *sklearn model selection* library was used to partition the data into training data (70%) and test data (30%) by using a simple random split method. The training data were used to build a predictive model using decision tree, random forest, naïve Bayes, SVM, and kNN classifiers. After building the predictive model, the inputs, including TB_Status, age, and weight, were computed to predict if the rat would hit the sample or not. Thereafter, the predictive models were evaluated for their prediction performance using accuracy, sensitivity, specificity, and recall metrics.

**Algorithm for the prediction of rat tuberculosis detection performance.**

I. Import and normalize the dataset (.csv)

II. Calculate IQR, mean, SD, minimum, and maximum

III. Perform splitting of the data set

1. if splitting is successful and not any constraints then

- train the model

2. Perform machine learning (ML) modeling based on decision tree, random forest, naïve Bayes, support vector machine, and k-nearest neighbor

3. Perform validation of the ML modeling

4. Perform ML model prediction

5. Validate the prediction model by calculating *accuracy*, *sensitivity*, *specificity*, and *F1 score*

6. if *accuracy and other parameters are good* then

- input: *TB_Status*, *age*, *weight*

7. Perform ML model prediction if the rat would hit the sample or not

8. Update the predicted value of new data for reporting

9. Make data visualization in Python

10. else

11. Perform termination check

12. else

13. End

## Discussion

### Principal Findings

The aim of this study was to build on the prior work of Jonathan et al [10] to develop models that predict if a trained TB-detection rat would hit (indicate the presence of TB within) a patient sample or not using ML techniques by incorporating variables related to the diagnostic results of the TB samples. This study used decision tree, random forest, naïve Bayes, SVM, and kNN ML techniques to build predictive models. The ML techniques successfully categorized the data by assigning a label to each computed data point. The results revealed that for the 5 different algorithms used, the classification accuracy was the greatest for SVM, suggesting its superiority to the decision tree, random forest, naïve Bayes, and kNN classifiers. The SVM classifier outperformed by yielding a classification accuracy of about 83.39% for predicting if the rat would hit the sample or not. This level of accuracy surpasses the 78.82% accuracy found with decision tree and naïve Bayes by Jonathan et al [10], suggesting that the inclusion of sample information serves as a valuable variable that influences the performance of TB-detection rats and improves the accuracy of the prediction models. Moreover, Jonathan et al [10] employed a small amount of data compared to the data used in this study. In fact, TB_Status was found to be the most significant variable in predicting rat TB detection performance. However, there was an insignificant accuracy difference between the constructed models and those created by Jonathan et al [10], which could be due to the characteristics of the data [16]. Therefore, the additional variables are likely to influence rat behavior, and the true status of patient samples can only be determined by available diagnostics.

### Conclusion

This study has shown the usefulness of ML techniques to identify factors that influence TB detection performance of rats. The techniques used were decision tree, random forest, naïve Bayes, SVM, and kNN to develop models that predict if the rat would hit the sample or not by incorporating valuable variables related to TB detection performance of rats. The performance of the predictive models was measured by accuracy, sensitivity, specificity, and F1 score metrics. The results showed that the SVM predictive model outperformed in the classification and prediction of the performance of rats in TB detection by yielding the highest accuracy of 83.39%. Furthermore, the obtained results suggest that the inclusion of variables related to the diagnostic results of TB samples improves the performance of the predictive models. Therefore, the results might benefit TB-detection rat trainers and TB decision-makers in improving the diagnostic accuracy of rats by predicting if a trained TB-detection rat would hit a patient sample or not. They can adopt several measures, including ensuring that all hit samples are confirmed within APOPO’s laboratory (ID_BL_APOPO). Furthermore, taking into consideration that the age of the rat at hit and clinic diagnostic results are predictors of detection performance.

## Appendix

Multimedia Appendix 1 Distribution of continuous independent variables.

Multimedia Appendix 2 Random forest variable importance plot.

## Abbreviations

APOPO: Anti-Persoonsmijnen Ontmijnende Product Ontwikkeling
DOTS: directly-observed treatment, short-course
FN: false negative
FP: false positive
kNN: k-nearest neighbor
ML: machine learning
SDT: Signal Detection Theory
SUA: Sokoine University of Agriculture
SVM: support vector machine
TB: tuberculosis
TN: true negative
TP: true positive
WHO: World Health Organization
