# Supplementary figures and images for: Machine Learning for Prediction of Tuberculosis Detection: Case Study of Trained African Giant Pouched Rats
Source: Online J Public Health Inform. 2024 Apr 16;16:e50771. doi: 10.2196/50771 (PMC11061786; doi:10.2196/50771)

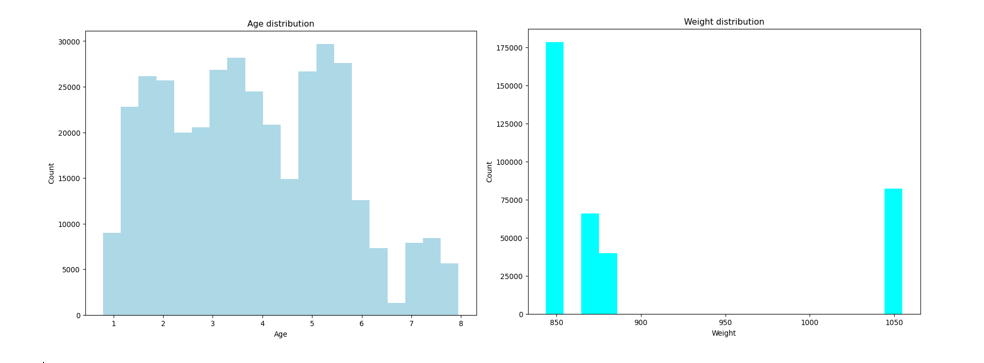

Supplement: Multimedia Appendix 1 [file ojphi_v16i1e50771_app1.png]

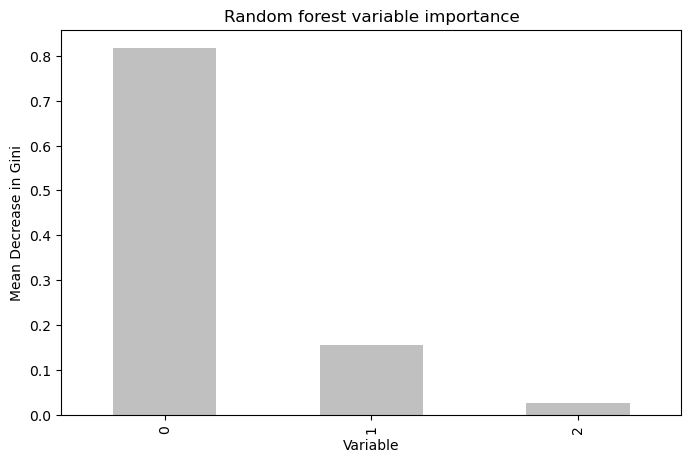

Supplement: Multimedia Appendix 2 [file ojphi_v16i1e50771_app2.png]
